# Brg1-mediated Nrf2/HO-1 pathway activation alleviates hepatic ischemia–reperfusion injury

**DOI:** 10.1038/cddis.2017.236

**Published:** 2017-06-01

**Authors:** Mian Ge, Weifeng Yao, Dongdong Yuan, Shaoli Zhou, Xi Chen, Yihan Zhang, Haobo Li, Zhengyuan Xia, Ziqing Hei

**Affiliations:** 1 Department of Anesthesiology, Third Affiliated Hospital, Sun Yat-sen University, Guangzhou, Guangdong 510630, China; 2 Department of Anesthesiology, Li Ka Shing Faculty of Medicine, The University of Hong Kong, Hong Kong, China; 3 Department of Medicine, State Key Laboratory of Pharmaceutical Biotechnology, University of Hong Kong, Hong Kong, China; 4 Department of Anesthesiology, Affiliated Hospital of Guangdong Medical University, Zhanjiang, Guangdong, 524001, China

## Abstract

Cytoprotective gene heme oxygenase 1 (HO-1) could be induced by nuclear factor E2-related factor 2 (Nrf2) nuclear translocation. The purpose of this study was to determine the role of Brahma-related gene 1 (Brg1), a catalytic subunit of SWI2/SNF2-like chromatin remodeling complexes, in Nrf2/HO-1 pathway activation during hepatic ischemia–reperfusion (HIR). Our results showed that hepatic Brg1 was inhibited during early HIR while Brg1 overexpression reduced oxidative injury in CMV-Brg1 mice subjected to HIR. Moreover, promoter-driven luciferase assay showed that overexpression of Brg1 by adenovirus transfection in AML12 cells selectively enhanced HO-1 gene expression after hypoxia/reoxygenation (H/R) treatment but did not affect the other Nrf2 target gene NQO1. Furthermore, inhibition of HO-1 by the selective HO-1 inhibitor zinc protoporphyria could partly reverse the hepatic protective effects of Brg1 overexpression while HO-1-Adv attenuated AML12 cells H/R damage. Further, chromatin immunoprecipitation analysis revealed that Brg1 overexpression, which could significantly increase the recruitment of Brg1 protein to HO-1 but not NQO1 promoter, was recruited by Nrf2 to the HO-1 regulatory regions in AML12 hepatocytes subjected to H/R. In conclusion, our results demonstrated that restoration of Brg1 during reperfusion could enhance Nrf2-mediated inducible expression of HO-1 during HIR to effectively increase antioxidant ability to combat against hepatocytes damage.

Hepatic ischemia–reperfusion (HIR) injury occurs inevitably during liver transplantation, trauma, hemorrhagic shock and other systemic low-flow diseases such as sepsis, respiratory failure and congestive heart failure.^1, 2, 3, 4^ HIR features in excessive production of reactive oxygen species (ROS) from various sources, leading to disturbance of the oxidation–antioxidation balance.^5, 6^ Excessive free heme, which is released from heme proteins under oxidative conditions, may be a major threat because it can catalyze the overproduction of ROS.^7^

Antioxidant enzyme heme oxygenase (HO-1), a rate-limiting enzyme in heme degradation,^8^ is highly inducible by a variety of discriminating stimuli inducing hepatic oxidative stress.^9, 10^ HO-1 transcription is modulated by an intertwined circuit in which nuclear factor E2-related factor 2 (Nrf2) plays an essential role.^11^ Nrf2 deficiency has been shown to exacerbate HIR injury and hepatocyte-specific Nrf2 overactivation provided protection against warm HIR.^12^ In resting cells, Nrf2 is retained in the cytoplasm as an inactive complex with Kelch-like ECH-associated protein 1 (Keap1).^13^ When cells are exposed to electrophiles or other reactive species, such as ROS, Nrf2 is released from the complex and translocate from cytoplasm into the nucleus. Once migrated to the nucleus, Nrf2 forms heterodimers with small Maf proteins and subsequently binds to the cis-acting antioxidant response element (ARE) within the gene promoters including HO-1 and NQO1.^14, 15^ The binding leads to transcriptional activation of a battery of genes that encode an array of phase II detoxifying or antioxidant enzymes, such as HO-1 and NQO1, as well as other cytoprotective proteins.^16, 17, 18^

Brahma-related gene 1 (Brg1) is the core ATPase in the SWI/SNF complex, which plays a central role in the activation and transcription of genes in mammalian cells.^19^ Brg1 has been reported to participate in the transactivation of proinflammatory mediators in macrophages treated with lipopolysaccharide.^20^ Interestingly, some recent studies indicated that Brg1 upregulation could also exert an antioxidative effect.^21^ Moreover, study showed that small interfering RNA knockdown of Brg1 in colon cancer cell SW480 selectively decreased inducible expression of *HO-1* gene after diethylmaleate treatment *in vitro*.^22^ However, whether overexpression of Brg1 could enhance Nrf2-mediated HO-1 gene transcription in hepatocyte subjected to ischemia/hypoxia and subsequent reperfusion/reoxygenation *in vivo* or *in vitro* in the epigenetic machinery remained unknown, if so, overexpression of Brg1 maybe a potential therapy in liver diseases involving ischemia–reperfusion.

Therefore, we sought to determine whether or not overexpression of Brg1 may have antioxidative effect against the hepatic damage during HIR, and if so, whether it functions via enhancing Nrf2-mediated HO-1 gene transcription in hepatocyte through epigenetic modification by Brg1.

## Results

### Ischemia–reperfusion induced liver pathological and oxidative stress

**Figure 1:**
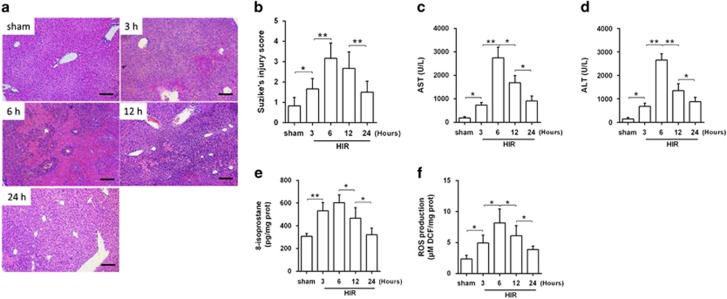
HI/R-induced liver damage. Animals were subjected to 70% liver warm ischemia for 60 min (min) and live tissues were collected at indicated time points. (**a**) Liver pathology was detected by H&E staining of liver paraffin sections. Representative H&E staining images are shown. (**b**) Suzike’s injury score was used as criteria to evaluate the injury degree of liver injury. (**c** and **d**) Liver function was evaluated by detection of serum AST and ALT concentration. (**e**) ELISA analysis showed 8-isoprostane level was elevated in response to HIR injury. (**f**) ROS production in liver after HIR was measured by fluorescence intensity of DCF using a fluorescence spectrophotometer. Each bar represents the mean±S.E.M. (*n=6* per group). *P<0.05, **P<0.01, one-way ANOVA with Tukey test

Mice were subjected to 70% hepatic ischemia for 60 min, followed by 3, 6, 12 or 24 h of reperfusion. As shown in Figure 1, compared to mice in the sham group, mice receiving HIR displayed collapse of hepatic architecture that was associated with severe congestion, intracellular edema and necrosis, resulting in significantly elevated total histopathological Suzuki’ score (*P*<0.05, Figures 1a and b), which maximized at 6 h after the onset of reperfusion. These pathological changes were corresponded to liver functional changes, evidenced by increases of serum aspartate amimotransferase (AST) and alanine aspartate amimotransferase (ALT) levels after reperfusion and peaked at 6 h after reperfusion (*P*<0.05, Figures 1c and d). These patterns of pathological injury were mirrored with significant elevation of oxidative stress in the liver, manifested by enhanced 8-isoprostane formation and increased ROS production (Figures 1e and f). The above results indicated that HIR induced serious liver injury during reperfusion as early as 6 h after reperfusion, in which oxidative stress may play a critical role.

### Hepatic Brg1 was decreased during early liver ischemia–reperfusion

**Figure 2:**
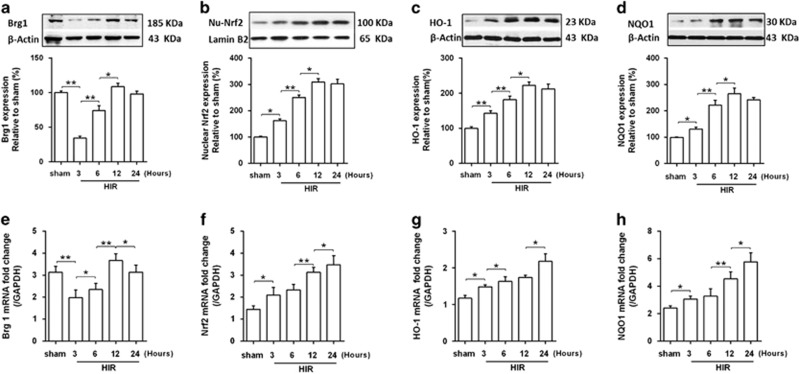
Expressions of Brg1, Nrf2 and Nrf2 downstream genes in the liver after hepatic I/R. (**a**, **b**, **c** and **d**) Western blot analysis showed that Brg1, nuclear Nrf2, HO-1 and NQO1 protein expressions were elevated in response to HIR in the liver at indicated time points. Representative images from one of three independent experiments were shown. Quantitative analyses of the results were also performed. (**e**, **f**, **g** and **h**) Transcript levels of Brg1, Nrf2, HO-1 and NQO1 in the liver in sham and HIR group were measured by RT-PCR. Each bar represents the mean±S.E.M. (*n=6* per group). *P<0.05, **P<0.01, one-way ANOVA with Tukey test

To explore the role of Brg1 in HIR, the changes of Brg1 were determined during the progression of HIR injury. Compared with the sham group, both mRNA and protein expressions of Brg1 were decreased at 3 h after HIR, which gradually recovered from 12 to 24 h after HIR (Figures 2a and e). Furthermore, the changes of mRNA and protein expression of Nrf2, the possible target of Brg1, and its downstream targets, HO-1 and NQO1 were detected. Interestingly, Nrf2 (Figures 2b and f) and antioxidant enzyme HO-1 and NQO1 (Figures 2c, d, g and h) were gradually elevated from 3 h after HIR and peaked at 24 h after HIR.

### Brg1 overexpression reduced oxidative injury during HIR

**Figure 3:**
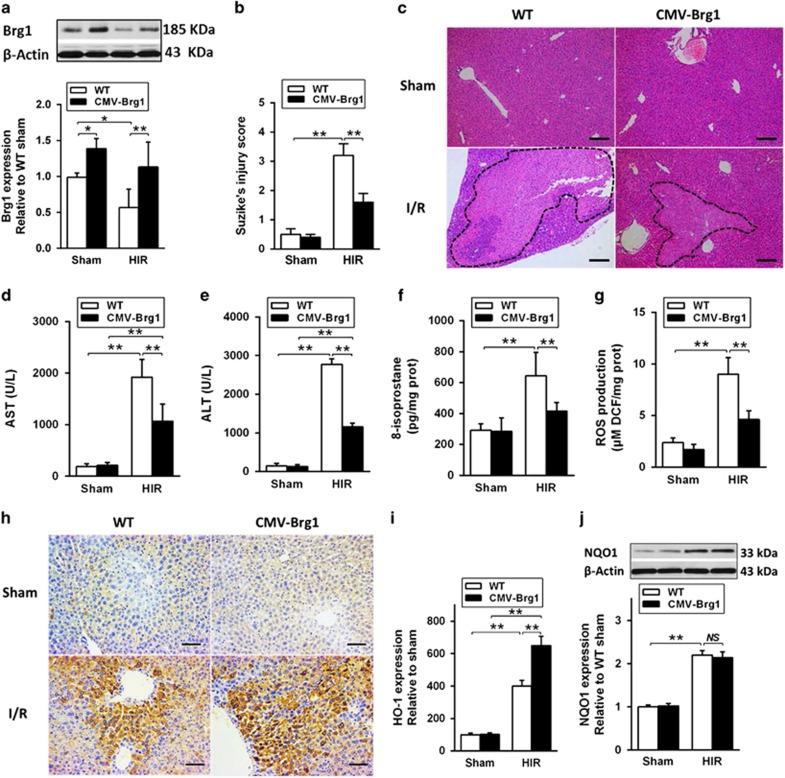
Overexpression of Brg1 attenuated HIR injury via enhancing antioxidant enzyme. Animals were subjected to 70% liver warm ischemia for 60 min and live tissues were collected at 6 h after reperfusion. (**a**) Western blot analysis showed that Brg1 expression was increased in Brg1 overexpression (CMV-Brg1) mice compared to WT mice both in the sham and HIR groups. (**b**) Suzike’s injury score showed lower scores in CMV-Brg1 mice than in WT mice after HIR injury. (**c**) Representative H&E staining images of liver collected from WT and CMV-Brg1 mice in the sham and HIR groups are shown. (**d** and **e**) Serum AST and ALT concentration showed an improved liver function in CMV-Brg1 mice after HIR injury. (**f**) ELISA analysis showed elevation of 8-isoprostane level was attenuated in CMV-Brg1 mice in response to HIR injury relative to that in the control. (**g**) ROS production measured by fluorescence intensity of DCF was reduced in CMV-Brg1 mice after HIR injury. (**h**) Immumohistochemical staining showed that liver HO-1 protein expression was elevated in response to HIR in CMV-Brg1 mice. (**i**) Quantitative analyses of the results from **h** were also performed. (**j**) Western blot analysis showed that liver NQO1 protein expression did not significantly change in CMV-Brg1 mice compared to WT mice. Each bar represents the mean±S.E.M. (*n=6* per group). *P<0.05, **P<0.01, one-way ANOVA with Tukey test. Imaginary line indicated the edge of apoptosis liver tissue

Brg1 was reduced after HIR that was associated with enhanced hepatic oxidative stress and liver injury, indicating a critical role of Brg1 in HIR. Thus, transgenic mice with Brg1 overexpression, cytomegalovirus (CMV)-Brg1 mice, were employed and subjected to HIR *in vivo* to examine whether or not liver with overexpression of Brg1 can attenuate HIR. We performed 60 min of ischemia and 6 h of reperfusion or sham operation in both wild-type and CMV-Brg1 mice. As shown in Figure 3, high expression of Brg1 protein and mRNA (Figure 3a) were confirmed in CMV-Brg1 mice. After 60 min of ischemia and 6 h of reperfusion, compared to wild-type (WT) mice, HIR injury was significantly attenuated in CMV-Brg1 mice evidenced by reduced Suzuki’ score, hepatic congestion, intracellular edema and necrosis (Figures 3b and c), accompanied with improved liver function manifested by lower serum AST and ALT levels (Figures 3d and e). Similar trend was observed in hepatic oxidative stress, compared with WT mice, Brg1 overexpression dramatically decreased post-HIR hepatic oxidative stress, evidenced by decrease of hepatic 8-isoprostane formation (Figure 3f) and ROS production (Figure 3g) (*P<*0.01). Interestingly, the expression HO-1 (Figures 3h–i) but not NQO1 (Figure 3j) in the liver was also elevated significantly in CMV-Brg1 mice subjected to HIR (*P*<0.01 *versus* WT HIR group). These results suggested that overexpression of Brg1 could attenuate liver damage induced by HIR by effectively reducing liver oxidative stress.

### Brg1 reduced HIR-induced hepatic oxidative stress by enhancing Nrf2 and HO-1

**Figure 4:**
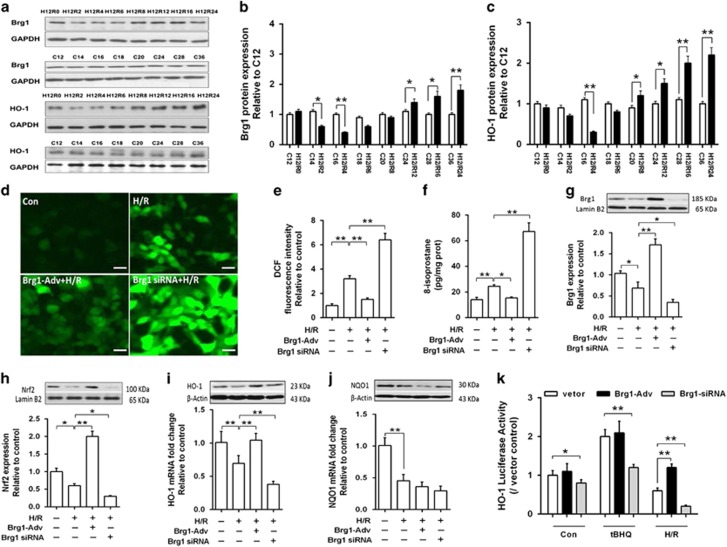
The role of Brg1 in AML12 cells subjected to H/R injury. (**a**) Western blot analysis showed the cellular Brg1 and HO-1 protein expression in AML12 hepatocytes subjected to hypoxia for 12 h and reoxygenation for 0 (H12R0), 2 (H12R2), 4 (H12R4), 6 (H12R6), 8 (H12R8), 12 (H12R12), 16 (H12R16) and 24 h (H12R24) before sample collection in comparison with the cells cultured for the same time as control (namely, C12, C14, C16, C20, C24, C28 and C36, representing 12–36 h of culture). The proteins from the H/R groups and from the control groups were loaded in the same gel when performing western blotting assay and displayed in parallel to facilitate comparison. Representative images from one of three independent experiments were shown. (**b** and **c**) Quantitative measurement of band intensity in **a** by densitometry analysis. (**d**) Fluorescence immunostaining of DCF in cells with Brg1 overexpression using Brg1-Adv transfection, and elevated in cells with Brg1 knockdown using Brg1-siRNA transfection during H/R (H12R4) injury. Representative images from one of three independent experiments were shown. (**e**) Bar graph showing the change in DCF fluorescent intensity. (**f**) ELISA assay showed that 8-isoprostane level was decreased after Brg1-Adv treatment and increased by Brg1-siRNA transfection during H/R (H12R4) injury. (**g** and **h**) Western blot analysis showed the change of Brg1 and Nrf2 protein expression in AML12 cells, respectively, under condition of Brg1 overexpression or knockdown. Representative images were shown and quantitative measurements were performed. (**i** and **j**) Western blot and RT-PCR analysis showed the protein and mRNA level of HO-1 and NQO1 in response to Brg1 overexpression or knockdown during cell H/R (H12R4) injury. (**k**) HO-1 promoter-driven luciferase activity assay was performed and tBHQ (20 *μ*M) was used as Nrf2 nuclear translocation positive control. Data are mean±S.E.M. of three independent experiments each performed in triplicate. *P<0.05, **P<0.01, one-way ANOVA with Tukey test

Study showed that in response to oxidative stress, Brg1 interacts with Nrf2 to mediate HO-1 induction.^22^ To determine the role of Nrf2 and HO-1 in Brg1-mediated attenuation of HIR injury, we established an *in vitro* cell model by subjecting AML12 hepatocytes to hypoxia/reoxygenation (H/R), which closely mimicked the stimuli and the effects of *in vivo* HIR and could effectively enhance Brg1 expression with adenovirus infection and reduce Brg1 by gene silence. As shown in Supplementary Figure S1, AML12 cells were subjected to 4, 8 or 12 h of hypoxia, followed by 2, 4, 6 or 8 h of reoxygenation. Significant cell injury (reduced cell viability and increased lactate dehydrogenase (LDH)) was observed when cells subjected to 12 h of hypoxia and 4 h of reperfusion (Supplementary Figures S1A–B). Also, we found both Brg1 and HO-1 protein expression were decreased in the early stage (0–6 h) of H/R and then gradually elevated from 8 h after cells reoxygenation (Figures 4a–c). We postulated that the reduction of Brg1 at early state of H/R played an important role in regulating HO-1 expression, and that restoring early HO-1 expression via Brg1 overexpression may attenuate hepatocytes H/R damage. Subsequently, we chose the model of hypoxia 12 h/reoxygenation 4 h, a time point at which both Brg1 and HO-1 protein expression reached their minimum level to explore the underlying mechanism. Thus, 12 h of hypoxia and 4 h of reoxygenation was chosen for our ensuring experiments.

As shown in Figure 4, H/R significantly increased hepatocytes oxidative stress evidenced as increased ROS production (Figures 4d and e) and elevated 8-isoprostane formation (Figure 4f), which was associated with decreased Brg1 expression (Figure 4g), enhanced protein expression of Nrf2 (Figure 4h) but reduced mRNA and protein expression and promoter luciferase activities of HO-1 and NQO1 (Figures 4i–k). Brg1 overexpression attenuated post-H/R oxidative stress accompanied by enhanced protein expression of Nrf2 as well as increased HO-1 induction and promoter luciferase activity. All these changes were canceled by Brg1 gene knockdown. Interestingly, Brg1 overexpression had no impact on H/R-induced reduction mRNA and protein expression and promotor luciferase activity of NQO1.

### HO-1 inhibition partly reversed the protective effects of Brg1 in HIR

**Figure 5:**
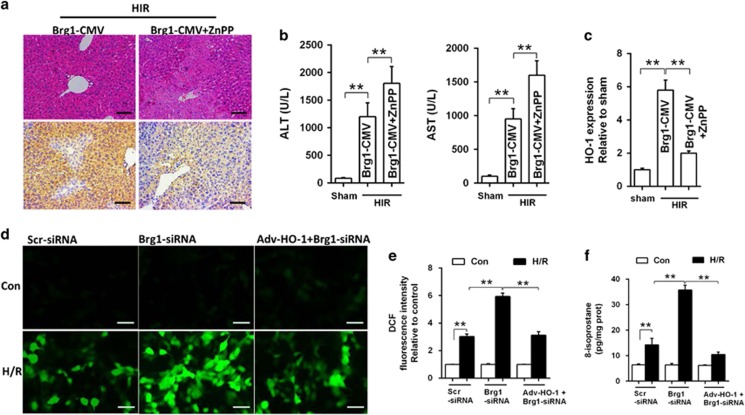
Inhibition of HO-1 reversed the protective effects of Brg1 overexpression. CMV-Brg1 transgenic mice subjected to HIR with or without HO-1 inhibitor ZnPP. Animals were killed at 6 h after reperfusion onset. (**a**) Liver pathology was detected by H&E staining and HO-1 expression was measured by immunohistochemical staining. Representative images from one of three independent experiments were shown. (**b**) The effects of HO-1 inhibition on Brg1-CMV mice post-HIR liver function were examined by AST and ALT. (**c**) Quantitative measurement of HO-1 immunohistochemical staining density in **a** by densitometry analysis. (**d** and **e**) Furthermore, AML12 cells injury was attenuated by overexpression of HO-1 in hepatocytes subjected to H/R (H12R4). Cell DCF fluorescence was detected and relative DCF fluorescence intensity was assayed. Representative images from one of three independent experiments were shown. (**f**) Cell 8-isoprostane was detected by ELISA assay to show the cellular oxidative stress level. Data are mean±S.E.M. of three independent experiments each performed in triplicate. *P<0.05, **P<0.01, one-way ANOVA with Tukey test. Silencer negative control scrambled (Scr) siRNA was used as a control

To confirm the role of HO-1 in Brg1-mediated attenuation of HIR injury, HO-1 was inhibited with zinc protoporphyria (ZnPP) or overexpressed with adenovirus Adv-HO-1 in CMV-Brg1 mice or AML12 hepatocytes in the presence or absence of HIR or H/R. We found that HO-1 inhibition with ZnPP (Figures 5a and c) deteriorated the liver function as assessed by AST and ALT (Figure 5b) and pathological change (Figure 5a) in CMV-Brg1 mice subjected to HIR. Furthermore, HO-1 overexpression with Adv-HO-1 attenuated Brg1 gene knockdown-induced exacerbation post-H/R oxidative stress in AML12 hepatocytes manifested as increase of DCFH-DA fluorescence (Figure 5d) and elevation of 8-isoprostane level (Figure 5e). These results indicated that HO-1 inhibition could partly reverse the protective effects of Brg1 overexpression during HIR or hepatocytes H/R injury.

### Brg1 facilitated Nrf2 to promote HO-1 induction during H/R

**Figure 6:**
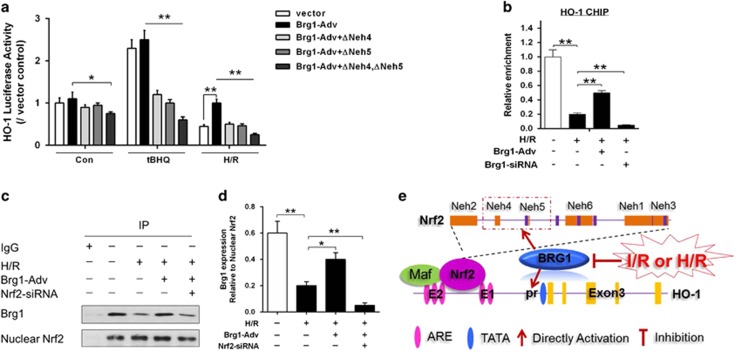
HO-1 promoter was regulated by Brg1/Nrf2 upon hepatocytes H/R. (**a**) AML12 cells were transfected with PGL3-HO-1-Luc, Brg1-Adv expression plasmids, Neh4 and/or Neh5 Nrf2 deletion mutants (△Neh4/△Neh5) without or with hypoxia for 12 h and reoxygenation for 4 h. Transfections and HO-1 promoter-driven luciferase assays were performed and tBHQ (20 *μ*M) was used as Nrf2 nuclear translocation positive control. (**b**) AML12 hepatocytes were then pretreated without or with Brg1-siRNA, or Brg1-Adv and then subjected to hypoxia for 12 h and reoxygenation for 4 h before sample collection. ChIP analyses were performed with antibodies against Brg1 and primers for the HO-1 promoter regions. (**c** and **d**) Furthermore, hepatocytes were pretreated without or with Nrf2 siRNA and Brg-Adv, then subjected to hypoxia for 12 h and reoxygenation for 4 h, Co-IP analysis were also performed with antibody against Nrf2. IgG was used as a negative control. Quantitative measurement of Brg1 band intensity was performed by densitometry analysis. (**e**) Diagram of HO-1 promoter activated by Brg1/Nrf2 upon H/R. Both human and mouse *HO-1* genes have two important distal enhancer regions, E1 and E2, located about 4 and 10 kbp upstream of the transcription start site. The dominant element in the E1 and E2 regions is the ARE, which mediates transcriptional activation in response to almost all HO-1 inducers tested. ARE represent binding sites of several transcription factors such as Nrf2. Under HIR condition, nuclear Brg1 interacts with Nrf2 via transactivation domain, Nrf2 ECH homology (Neh)4 and Neh5, which promotes Nrf2 binding to the ARE within the gene promoter of HO-1. Data are mean±S.E.M. of three independent experiments each performed in triplicate. *P<0.05, **P<0.01, one-way ANOVA with Tukey test

We have showed that Brg1-mediated attenuation of oxidative stress in hepatocytes subjected to H/R by enhancing Nrf2 and HO-1, in order to determine how HO-1 promoter was regulated by Brg1 and Nrf2 interaction, luciferase assay was performed. HO-1 luciferase activity was increased in AML12 cells after transfected with Brg1-Adv plasmid. tBHQ (20 *μ*M) was used as Nrf2 nuclear translocation positive control. H/R decreased HO-1 luciferase activity, which was restored by Brg1-Adv plasmid transfection. However, the Brg-1-Adv-induced HO-1 luciferase activity elevation was canceled by transfection with Neh4 and/or Neh5 Nrf2 mutants (△Neh4 and/or △Neh5) transfection in AML12 cells subjected to H/R (Figure 6a), suggesting that Brg1 may directly interact with Neh4/Neh5 Nrf2 domains to facilitate HO-1 gene expression. Next, to exposure whether Brg1 directly interacted with HO-1 promoter in this process, chromatin immunoprecipitation (ChIP) assay was applied in our current study. AML12 hepatocytes were pretreated with or without Brg1-siRNA or Brg1-Adv and then subjected to hypoxia for 12 h and reoxygenation for 4 h before sample collection. ChIP analysis was performed with the anti-Brg1 antibody and primers of HO-1 were used in this experiment. ChIP analysis revealed that the recruitment of Brg1 protein found in the HO-1 promoter was markedly reduced in response to Brg1-siRNA, and Brg1 overexpression could significantly increase the recruitment between Brg1 protein and HO-1 promoter (*P*<0.01 *versus* control) (Figure 6b).

To further explore the interplay between Nrf2 and Brg1 in H/R-induced transactivation of *HO-1* gene, we knocked down Nrf2 with siRNA in AML12 hepatocytes. Brg1 was immunoprecipitated with antibody conjugated to agarose beads, followed by immunoblotting with anti-Nrf2 antibodies. As shown in Figure 6c, we found that there existed a strong association/interaction between Brg1 and nuclear Nrf2, but this interaction was much weaker upon H/R. Brg1-Adv treatment could significantly promote Brg1/Nrf2 co-localization. However, siRNA targeting Nrf2 could reduce the co-localization of Brg1/Nrf2 (Figure 6d). Taken together, these results support a model wherein a Brg1/Nrf2 complex form on the HO-1 promoter in response to H/R to activate transcription in hepatocytes cells as described in Figure 6e.

## Discussion

Oxidative stress is triggered by ROS released from HIR or H/R. Nrf2 activation and the downstream antioxidant enzyme upregulation during the early stage of HIR could promote the functional recovery of the impaired liver. The key to this process is the transactivation of antioxidant enzyme mediated by Nrf2 in response to HIR. Here we report that epigenetic factors Brg1 contributes to Nrf2-mediated HO-1 gene transactivation, which is critically involved in the pathogenesis of HIR injury.

Ischemia/reperfusion injury is characterized as a pathophysiologic process whereby hypoxic organ damage is accentuated following return of blood flow and oxygen delivery.^23^ One of the principal causes of HIR injury is the inherent oxidative damage that occurs during reperfusion inflicted by the generation of ROS.^24^ The extent of early ROS formation is critical to the magnitude of the final tissue injury and enhancing hepatic antioxidant capacity has been proven to be effective in reducing HIR injury.^25^ Promoting the transcription efficiency of antioxidant enzyme may be potentially a novel promising therapeutic option for HIR injury.^26^ Nevertheless, the approach to enhancing transcription efficiency is still lacking.

Gene transcription is tightly regulated at different levels to ensure that the transcriptome of the cell is appropriate for developmental stage and cell type.^27^ The chromatin state in which a gene is embedded determines its expression level to a large extent.^28^ Activation of transcription is typically accomplished by the recruitment of chromatin-associated multi-subunit protein complexes including SWI/SNF.^29^ As the core ATPase of SWI/SNF, Brg1 is essential *in vivo*, suggesting that Brg1 containing SWI/SNF nucleosomal remodeling complexes are critical in mammalian organisms.^19^ Brg1 regulates chromatin structure in response to stress, and we found that Brg1 was gradually increased in the liver during the early stage (6–24 h) of reperfusion after a transient decrease at the onset of reperfusion (before 3 h). By using the CMV-Brg1 transgenic mice in which Brg1 was overexpressed, we found that Brg1 overexpression could effectively reduce the oxidative stress occurred during the reperfusion period. This is a novel finding, since most studies have merely and controversially shown that Brg1-dependent pathway connects the epigenetic regulation of proinflammatory genes rather than antioxidant genes to the pathogenesis of inflammation disease.^30^ However, the mechanism of Brg1 antioxidant activity in HIR is unclear.

Nrf2 is a stress-sensing genetic transcription factor, which appears to be a master regulator of cellular responses to oxidative damage and other stressful conditions. The Nrf2 antioxidant response pathway is ‘the primary cellular defense against the cytotoxic effects of oxidative stress’.^31^ Strategies that can effectively activate transcription factor Nrf2 and promote the downstream antioxidant enzyme genes transcription may lead to better outcome during HIR.^32^ It is well known that ROS can activate signal transducing molecules through the effects on oxidation-prone cysteine-rich domains, thereby activating gene transcription, and we found that Nrf2 was activated during liver oxidative injury in HIR. However, the production of Nrf2 downstream antioxidant HO-1 and NQO1 was insufficient to reduce oxidative stress and unable to curb hepatic damage amplification. In addition, we found that chromatin remodeling factor Brg1 was suppressed both *in vitro* and *in vivo* during early HIR and that overexpression of Brg1 could promote Nrf2 transcription and dramatically induce the downstream enzyme HO-1 gene expression, which was consistent with other observation which showed that Brg1 could modulate the expression of alpha interferon-inducible gene through interactions with specific transcription factor STAT2.^33^ Despite that inflammation mediated by upregulation of Brg1 induced endothelial injury in the pathogenesis of atherosclerosis,^27^ the Nrf2/HO-1 pathway activated by Brg1 upregulation in the present study was found to protect the liver against HIR injury.

Under basal conditions, the physiological low-level expression of HO-1 functions to maintain redox hemeostasis, cooperating with other antioxidant enzymes. However, no apparent histological abnormalities were observed in hepatocyte-specific conditional HO-1 gene knockout mice under normal condition.^34^ In contrast, the inducible expression of HO-1 was thought to be more important than its basal expression for hepatic HO-1 functions. Induction of HO-1 expression involves two fundamental regulatory pathways either via a heme-dependent or a heme-independent mechanism.^35^ Despite the differences in the two pathways, the effects of the diverse factors on hepatic HO-1 gene expression appear to be controlled mainly at the transcriptional level. *HO-1* genes have two important distal enhancer regions, E1 and E2.^36^ The dominant element in the two regions is the stress-responsive element, which mediates transcriptional activation in response to almost all HO-1 inducers tested (Figure 4e). In our study, although inducible HO-1 has an about 80% increase during the first 3–6 h of HIR, the oxidative stress/antioxidant balance was still inclined to oxidative stress and liver damage occurred, which were associated with decreased Brg1 expression, suggesting that the decrease of Brg1 may have played a critical role in limiting Nrf2/HO-1 expression to a higher level to combat against hepatic oxidative stress and HIR. And this hypothesis was tested using the CMV-Brg1 mice in which hepatic Brg1 was overexpressed and Nrf2/HO-1 pathway was activated and HIR injury was reduced. These results indicated that, in order to combat HIR, a level of HO-1 higher than that in the early phase of HIR (3–6 h) is needed,which is regulated by Brg1. However, as the regulation of HO-1 gene expression in the intact liver was complex and there were different cell types in liver which may affect the expression of HO-1 in different manners, we then conducted *in vitro* experiments and investigated the role of Brg1 on Nrf2/HO-1 in AML12 hepatocytes to further explore the mechanism of Brg1 acts on Nrf2/HO-1 pathway. Interestingly, we found that in AML12 hepatocytes, Brg1 was reduced during the early phase of reoxygenation (2–6 h), which is in parallel with the reduction of HO-1 during hypoxia and reoxygenation, indicating that our *in vitro* model of hypoxia reoxygenation may mimic the very early phase (earlier than 3 h) of HIR in mice, a stage that both Brg1 and HO-1 were reduced in response to HIR.

In addition, we found that Brg1 was selectively recruited to *HO-1* but not *NQO1* gene during HIR injury, leading to a different induction of these two genes through interaction with Nrf2, and these phenomena were also observed in SW480, SW13 and 293 T cells in the study of Zhang *et al*,^22^ which showed that knockdown of Brg1 in SW480 cells selectively decreased the inducible expression of *HO-1* gene after diethylmaleate treatment. We also found that Brg1 activated *HO-1* gene in hepatocytes in oxidative stress state instead of normal state in which Nrf2 was inactivated indicating that activation of Brg1 overexpression and Nrf2 activation could jointly promote HO-1 gene expression in ischemia–reperfusion condition. These were also observed in our previous study, which showed that adiponectin ameliorated hyperglycemia-induced cardiac hypertrophy and dysfunction by concomitantly activating Nrf2 and Brg1.^37^ Furthermore, we found that Nrf2 could recruit Brg1 to HO-1 promoter in HIR. The mechanism of Brg1-mediated Nrf2/HO-1 transcription has yet to be elucidated. One mechanism could be that Nrf2 independently bind to nucleosomal DNA or DNA between nucleosomes, and the subsequent binding of Brg1 leads to instability of neighboring nucleosome and thus sequester more Nrf2. This is a process of cascade reaction called reconstruction.^38^ Another possible mechanism may involve nucleosome sliding.^39^ To be specific, Brg1 independently binds to nucleosome without altering its structure, but unchain it from DNA to induce nucleosome sliding, which enables the binding of Nrf2, hereby stabilizing the regions without nucleosome.^40^ In Nrf2, there are six domains namely Neh1 (Nrf2 ECH homology 1) to Neh6 that have been identified and Neh4/Neh5 have been considered as transcription-related domains.^41^ In the current study, we found under HIR condition, nuclear Brg1 interacts with Nrf2 via transactivation domain, Nrf2 ECH homology (Neh)4 and Neh5, which promotes Nrf2 binding to the ARE within the gene promoter of HO-1. On the other hand, enhancing Brg1 activity could directly enhance the binding of Brg1 to HO-1 promoter, subsequently leading to HO-1 gene transcription (Figure 6e).

Of note, Brg1-mediated dynamic chromatin remodeling processes are required for the initial step in gene expression, which is regulated by epigenetic processes including DNA methylation,^42^ histone modifications^43^ and the action of small noncoding RNAs.^44^ On the basis of our current results, the above epigenetic modification changes will be involved in our further study. Moreover, we only mentioned that Brg1 upregulation protected hepatocytes from H/R damage, and more attention will be paid in the specific hepatocyte damage feature, including proliferation reduction, apoptosis or autophagy. In addition, as Brg1 also takes part in the syntheses of proinflammatory mediators,^45^ whether overexpression of Brg1 will present proinflammation effect in the late phase of reperfusion following HIR also needs to be investigated in the future.

**Figure 7:**
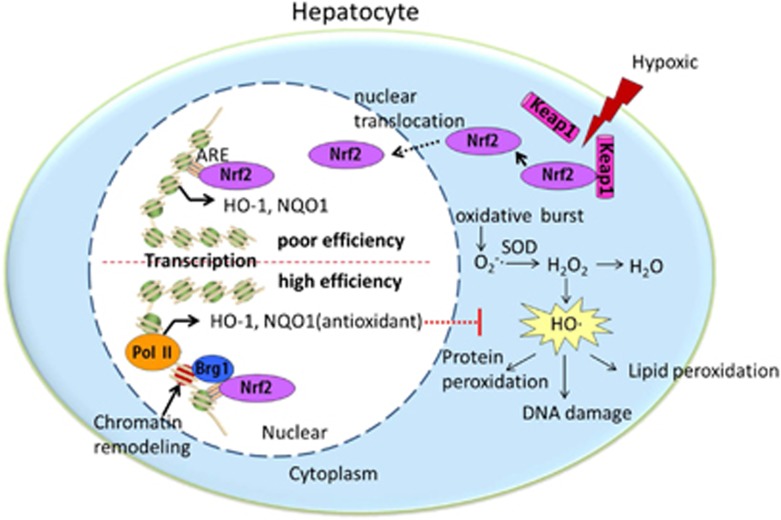
Proposed signaling of Brg1-mediated Nrf2/HO-1 pathway activation in HIR injury. Keap1 dissociation from Nrf2 leads to Nrf2 translocation from cytoplasm to nucleus during HIR injury. However, with poor efficiency, hepatocyte could not produce enough antioxidant HO-1, which is the downstream target gene of keap1/Nrf2 pathway. Upregulation of the chromatin remodeling factor Brg1 could enhance transcription factor Nrf2 binding to HO-1 DNA sequence and promote HO-1 generation in a high efficiency way. Antioxidant enzyme HO-1 then suppresses the free radical generated from oxidative burst during hepatocyte H/R injury

In summary, this study demonstrates that Brg1-mediated chromatin-remodeling activity is essential for Nrf2 transcription and the downstream antioxidant enzyme HO-1 gene induction during hepatic oxidative stress (Figure 7).

## Materials and Methods

### Antibodies and reagents

Antibodies recognizing Brg1 and Nrf2 were purchased from Abcam Company (Cambridge, MA, USA). Antibodies rose against HO-1 and NQO1 were from Santa Cruz Biotechnology (Dallas, TX, USA), while antibodies against Lamin B2 and *β*-actin were from Cell Signaling Technology (Danvers, MA, USA). HO-1 inhibitor ZnPP, Brg1 (NM_001174078.1) siRNA, Nrf2 (NM_010902.3) siRNA and control siRNA were obtained from Sigma-Aldrich (St. Louis, MO, USA). Recombinant adenoviruses containing Brg1 (Brg1-Adv, 1 × 10^10^ pfu/ml) or HO-1(NM_010442, HO-1-Adv, 1 × 10^10 ^pfu/ml) were designed and prepared from GeneCopoeia company.

### Transgenic mice

Brg1 transgenic mice were obtained from Cyagen Biosciences Inc. (Guangzhou, China). To obtain the CMV-Brg1 mice, first, the pRP.ExSi-CMV-Brg1 vector was constructed. *Brg1* gene was then overexpressed by using CMV promoter. Next, the pRP.ExSi-CMV-Brg1 vector was linearized and purified to get the plasmid, which could be used for microinjection. The pronuclei of fertilized eggs from hyperovulated C57BJ/6N were microinjected with this Brg1 DNA construct. The fertilized eggs with better state were chose to transplant into pseudopregnancy mother mouse from the institute of cancer research. Mice were bred and screened by Southern blot analysis of their tail DNA. Among six established lines (pRP.ExSi-CMV-Brg1-22, pRP.ExSi-CMV-Brg1-24, pRP.ExSi-CMV-Brg1-27, pRP.ExSi-CMV-Brg1-41, pRP.ExSi-CMV-Brg1-45 and pRP.ExSi-CMV-Brg1-51) of transgenic mice, two lines (ExSi-CMV-Brg1-24 and pRP.ExSi-CMV-Brg1-45) with substantial Brg1 protein expression were used for further experiments. Six different control mice and six different CMV-Brg1 mice were used in each related group for experiments. Genotype of Brg1-CMV mice were initially identificated by reverse transcription-PCR analysis and only phenotypes that were commonly observed were used in our current study. Brg1 transgene PCR primer forward: 5′-GCACCAAAATCAACGGGAC-3′, reverse: 5′-CTAGGACCCAGCATTG CAC-3′ Internal control PCR primer forward: 5′-ACTCCAAGGCCACTTATCA CC-3′, reverse: 5′-ATTGTTACCAACTGGGACGACA-3′. The materials about CMV-Brg1 transgenic mice were presented as supplemental document (Vector Building and Transgenic Products Report).

### Animals and hepatic warm ischemia–reperfusion model

C57BL/6 mice were obtained from SLAC Laboratory Animal Co. Ltd (Shanghai, China). Mice were raised and bred in Vaccine Institute Laboratory in the Third Affiliated Hospital of Sun Yat-Sen University. After a midline laparotomy incision, an atraumatic vascular clip was placed on the vessels blocking the portal venous and hepatic arterial blood supply to the median and left lateral lobes of the liver, which resulted in ~70% mouse liver I/R injury.^46, 47^ Some of the CMV-Brg1 mice were received ZnPP (i.p., 5 mg/kg) 2 h before surgery.^48^ All animal experiments were approved by the Animal Care and Used Committee of Sun Yat-Sen University (Guangzhou, China) and followed the ‘Guide for the Care and Use of Laboratory Animals’ (NIH Publications no. 8023, revised 1978) guidelines for the treatment of animals.

### Cell culture and H/R model

Mice hepatic cells AML12 (ATCC, Manassas, VA, USA) was maintained following the vendors’ recommendations. H/R was performed as described in our previous study.^49^ In brief, cells were placed in Galaxy 48 R hypoxia incubator (Eppendorf Company, Hamburg, Germany) with hypoxia gas mixture (5% CO_2_, 94% N_2_ and 1% O_2_) at 37 °C. And, following the completion of the corresponding hypoxia time, cells were then taken out from hypoxia incubator and placed in 5% CO_2_ incubator. Cell survival was assessed using the Cell Counting Kit-8 (CCK-8) and LDH assays.

### Liver injury assay

Histology analysis was performed as previously described.^50^ Briefly, the liver paraffin sections with hematoxylin and eosin (H&E) staining were observed under a light microscope by an investigator who was initially blinded to experimental groups, and five randomly selected fields of each slide were chose and analyzed, and injury score was graded according to the Suzike’s criteria in Table 1. Serum AST and ALT levels were determined by a 7180 Biochemical Analyzer (Hitachi, Japan). 8-Isoprostane (also named 15-F2t-Isoprostane) ELISA kits (Cayman Chemical Company, Ann Arbor, MI, USA) were used to detect the levels in the liver tissues.

### Plasmids, transfection and reporter assay

Recombinant adenovirus was generated by homologous recombination and amplified in HEK293 cells. The Brg1 or HO-1 recombinant adenovirus was, respectively, diluted with DMEM/F12 cell culture medium and added directly to the cells (MOI=10) at 50% confluence. After infection for 24 h, the cells were transfected with or without Brg1-siRNA or Nrf2 siRNA for 48 h and then H/R was induced as previously described.^50^ Plasmids encoding Nrf2 Neh4 and/or Neh5 mutants (Nrf2 with the Neh4/Neh5 domain deleted, obtained from GeneCopoeia company, Guangzhou, China) were also used in the *in vitro* experiments in AML12 cell line to selectively knockdown the domain of Nrf2. Silencer negative control scrambled (Scr) siRNA (Ambion Inc., Langhorne, PA, USA) was used as a control. Tertiary butyl hydroquinone (tBHQ, Sigma-Aldrich) was dissolved in DMSO and used as a positive Nrf2 activation control. Before use, the chemicals were dissolved in the culture media at a concentration of 20 *μ*M, keeping the final carrier concentration at 0.1%. Transient transfections were performed with Lipofectamine 2000 (Invitrogen, Carlsbad, CA, USA) according to the manufacturer’s protocol.

Luciferase assays were performed with a dual-luciferase reporter kit (Promega Luciferase Assay System E1501). The pre-processed AML12 cells lysis solution (20 *μ*l) was collected and added with 100 *μ*l luciferase assay reagent II working liquid. The luminous value (F) of firefly luciferin enzyme was determinate by GloMax bioluminescent detector. Then 100 *μ*l Stop & Glo reagent (Promega, Madison, WI, USA) was added to stop reaction and the luminous value (R) of renilla luciferase was detected. Relative luciferase activity was counted through F/R. The 3′UTR of the HO-1 and NQO1 were amplified by PCR using primers and cloned into the PGL3 luciferase reporter vector. HO-1 primers, forward 5′-TGCTAGCCTGGTGCAAGATA-3′ and reverse 5′-GCCAACAGGAAGCTGAGAGT-3′ NQO1 primers, forward 5′-GTCCATTC -CAGCTGACAACCA-3′ and reverse 5′-TTGCCCTGAGGCTCCTAATC-3′. The relative expression of the target genes were calculated using the 2^−ΔΔ^Ct method.^51^

### ROS production assay

The ROS production in mice liver tissue was detected using an OxiSelect intracellular ROS assay kit (Cayman Chemical Company). Sample homogenate supernatant was incubated with DCFH-DA (100 *μ*M) at 37 °C for 30 min, and the reaction was terminated by chilling the reaction mixture in ice. Using a fluorescence spectrophotometer (SpectraMax M5, Molecular Devices, Sunnyvale, CA, USA), the formation of the oxidized fluorescent derivative (DCF) was monitored at excitation and emission wavelengths of 480 nm and 530 nm, respectively. The free radical content was quantified using the generated DCF standard curve (Supplementary Figure 2A) and the calculated results were expressed as *μ*mol of DCF/mg protein. ROS in hydrogen peroxide-treated AML12 cells were also measured as control to ensure the selectivity of DCF oxidation (Supplementary Figure 2B). In *in vitro* study, AML12 cells were washed with ice-cold PBS and incubated with 100 *μ*M DCFH-DA for 30 min at 37 °C. Then, the medium was discarded and cells were washed with ice-cold PBS in the dark, and ROS generation was evaluated by the fluorescence intensity measured also by a fluorescence spectrometry and images were obtained with a florescence microscope (Olympus, IX51, Tokyo, Japan).

### RNA extraction and quantitative real-time PCR analysis

The RNA of liver tissue and cells were extracted following the manufacturer’s instruction (Omega, Norcross, GA, USA), and then the purity and integrality were detected as described in our previous study.^52^ The levels of mRNA were quantified through RT-PCR assay using SYBR Green Real-time PCR kit (Takara, Tokyo, Japan) in ABI PRISM 7500 Sequence Detection System. The primers and Taqman probe set for real-time reactions were as follows: Nrf2, forward 5′-ACAGTGCTCCTAT GCGTGAA-3′ reverse 5′-GAGCCTCTAAGCGGCTTGAA-3′ Brg1 forward 5′-AGATGGAGTAGCCCTTAGCA-3′ and reverse 5′-GAGGTCCCCTCTCTAGACA GTT-3′ HO-1, forward 5′-TGCTAGCCTGGTGCAAGATA-3′ and reverse 5′-GCCAACAGGAAGCTGAGAGT-3′ NQO1, forward 5′-GTCCATTCCAGCTGACAACCA-3′ and reverse 5′-TTGCCCTGAGGCTCCTAATC-3′ GAPDH, forward 5′-GGCCTCCAAGGAGTAAGAAA-3′ and reverse 5′-GCCCCTCC -TGTTATTATGG-3′. Data were normalized relative to GAPDH or control group.

### ChIP analysis

ChIP assays were implemented using ChIP kit (Millipore, Germany) according to the instruction with the use of anti-Brg1 (Abcam) antibody. DNA products from the immunoprecipitation were quantified by qRT-PCR relative to input. Precipitated genomic DNA was analyzed using Q-PCR method. PCR were performed against the HO-1 (primers forward 5′-GTGACCCGCGTACTTAAAG-3′ and reverse 5′-TCCACTCACTGGTTGTATGC-3′) or NQO1 (primers forward 5′-CCTGCTGCA GCTGATATTTC-3′ and reverse 5′-TGATGGATCTCAGTGGAGTCT-3′).

### Immunohistochemical assay for HO-1

Paraffin-embedded liver tissue wax blocks were sectioned at 5 *μ*m. After being dewaxed and rehydrated, the sections were incubated in 3% hydrogen peroxide/methanol. Heat-induced antigen retrieval was performed by heating in 10 mM sodium citrate buffer for 10 min. Sections were incubated in anti-Beclin1 antibody (Cell Signaling Technology) at 1:200 dilution at 4 °C overnight. 3,3′-Diaminobenzidine Substrate Chromogen System (Dako, Carpinteria, CA, USA) was employed during the detection procedure. Subsequently, the sections were counterstained using hematoxylin for 10 s. Finally, after being dehydrated in ethanol, cleared in xylene and mounted, the sections were observed in the light microscope by a pathologist who was initially blinded to treatment groups, and five random fields of each slide were semi-quantified and averaged using the software ImageJ 1.48 (National Institutes of Health, Bethesda, MD, USA) according to its instructions, then come up with the data of density of target protein positive cell, and the relative density (/sham) of protein represents the protein expression level of HO-1.

### Immunoprecipitation and immunoblotting

Cultured AML12 hepatocytes were homogenized in lysis buffer. A total of 500 *μ*g extracts were subjected to immunoprecipitation with 2 *μ*g Brg1 primary antibody or IgG as negative control in the presence of 20 ml protein A/G PLUS-Agarose. After extensive PBS washes, the immunoprecipitates were denatured with sodium dodecyl sulfate loading buffer and subjected to analysis for Brg1 and Nrf2 expression by western blot as described below.

### Protein extraction and western immunoblotting

Whole-cell lysates (Brg1, NQO1 and HO-1) and nuclear proteins (Nrf2) were performed as described in our previous study.^52^ Western blot analyses were performed with anti-Brg1 (1:1000), anti-Nrf2 (1:1000), anti-HO-1 (1:250), anti-NQO1 (1:250), anti-lamin B2 (1:2000) and anti-*β*-actin (1:2000) antibodies.

### Statistical analysis

Data are expressed as mean±S.E.M. Biochemical assays were performed in triplicate for each specific sample. Therefore, all the data points are means of numbers themselves resulting from means of triplicate measurements for these parameters. Significance was evaluated using one-way ANOVA test (SPSS 13.0, SPSS Inc, Chicago, III) followed by Tukey post hoc multiple comparisons test for unpaired values. *P*<0.05 was considered statistically significant.

## Figures and Tables

**Table 1:** Suzuki’s histological criteria

**Grade**	**Congestion (%)**	**Vacuolization (%)**	**Necrosis (%)**
0	None	None	None
1	Minimal (10)	Minimal (10)	Minimal (10)
2	Mild (11–30)	Mild (11–30)	Mild (11–30)
3	Moderate (31–60)	Moderate (31–60)	Moderate (31–60)
4	Severe (>60)	Severe (>60)	Severe (>60)
