# Supplementary figures and images for: Brg1-mediated Nrf2/HO-1 pathway activation alleviates hepatic ischemia–reperfusion injury
Source: Cell Death Dis. 2017 Jun 1;8(6):e2841–. doi: 10.1038/cddis.2017.236 (PMC5520895; doi:10.1038/cddis.2017.236)

**A**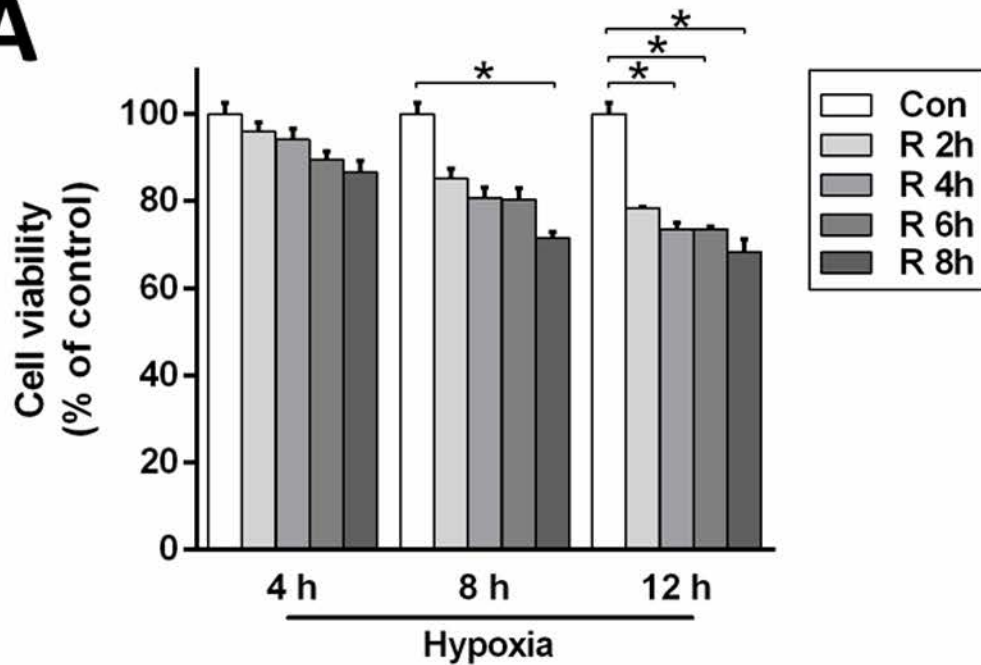**B**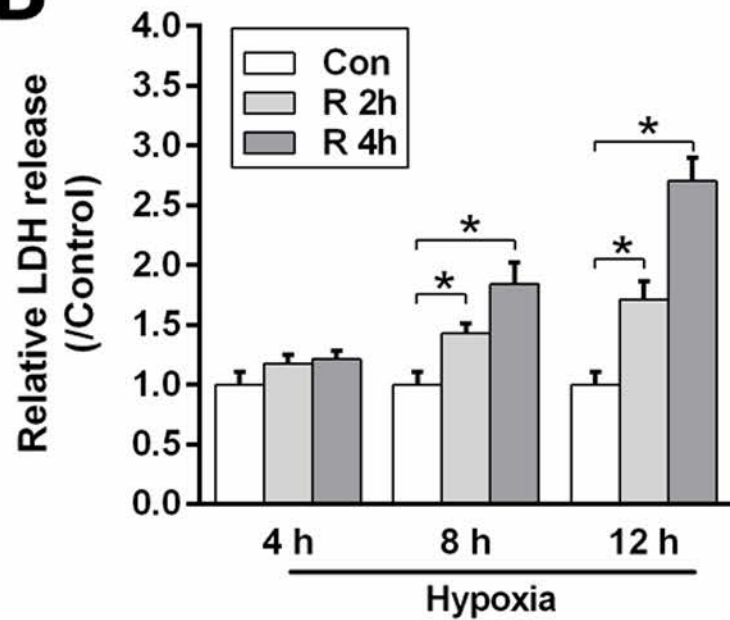

Supplement: Supplementary Figure S1 [file cddis2017236x1.pdf]

**A**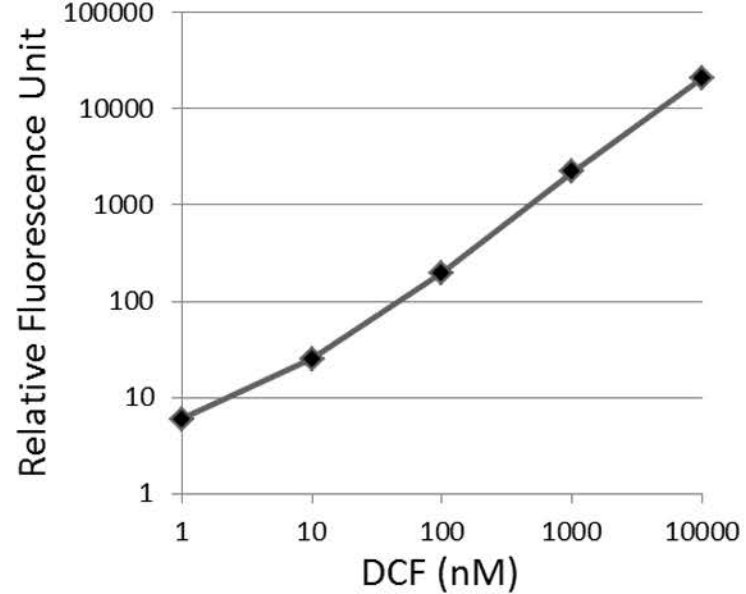**B**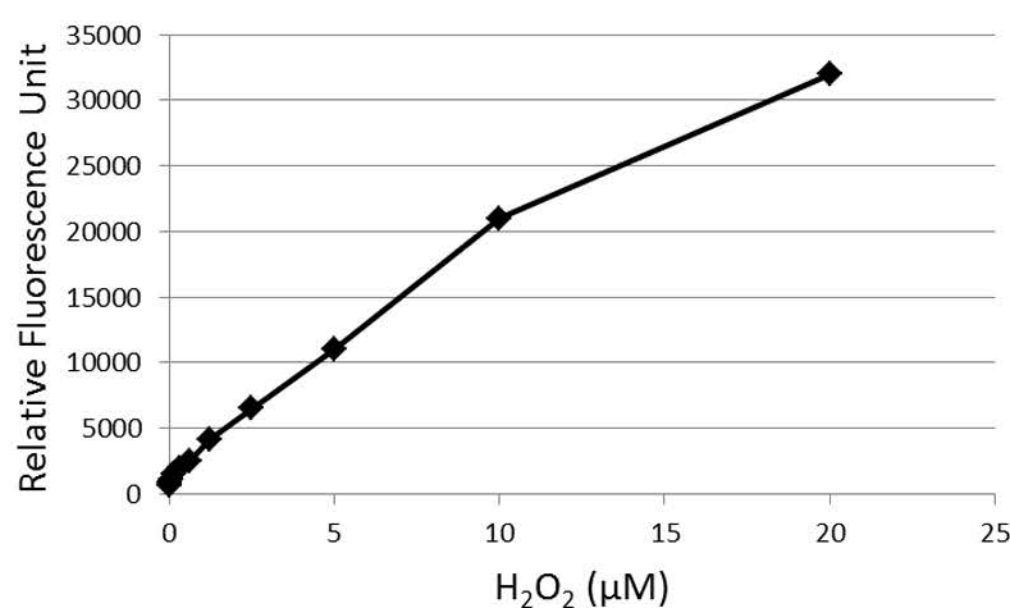

Supplement: Supplementary Figure S2 [file cddis2017236x2.pdf]
