# Supplementary material for: Brg1-mediated Nrf2/HO-1 pathway activation alleviates hepatic ischemia–reperfusion injury
Source: Cell Death Dis. 2017 Jun 1;8(6):e2841–. doi: 10.1038/cddis.2017.236 (PMC5520895; doi:10.1038/cddis.2017.236)
Supplement: Supplementary Figure Legends [file cddis2017236x3.docx]

**Supplementary Figure legends**

**Figure S1: AML12 cells hypoxia/reoxygenation (H/R) model was established.** (**A**) AML12 cells were subjected to 4, 8, or 12 hours of hypoxia, followed by 2, 4, 6, or 8 hours of reoxygenation. Cell viability was measured by CCK-8 assay. (B) Cell damage was detected by the concentration of LDH. Data are mean ± SEM of three independent experiments each performed in triplicate. **: p< 0.05*, ***: p< 0.01*, *one-way ANOVA* with *Tukey test*.

**Figure S2: DCF and Hydrogen Peroxide Standard Curve.** (**A**) A series of DCF standards in the concentration range of 1 μM-10 μM were diluted in cell culture media. Each DCF standard was transferred to a 96-well plate suitable for fluorescence measurement and the fluorescence was read to draw the DCF standard curve. (**B**) AML12 cells in a 96-well plate were first pretreated with 1 microM DCFH-DA for 60 min at 37ºC. Cells were then treated with various concentrations of H_2_O_2_ for 20 min. The fluorescence was read to draw the hydrogen peroxide standard curve.
